# Integrating One Health Into Health Systems: A Systematic Review and Narrative Synthesis of Implementation Challenges, Opportunities and Strategic Directions

**DOI:** 10.1002/puh2.70260

**Published:** 2026-04-28

**Authors:** Md. Shahidul Islam, Nusrat Jahan, Daniel Teshome Gebeyehu

**Affiliations:** ^1^ School of Health University of New England Armidale New South Wales Australia; ^2^ School of Veterinary Medicine Wollo University Dessie Ethiopia

**Keywords:** challenges, health systems, implementation, multisectoral collaboration, One Health, opportunities, systematic review

## Abstract

**Background:**

The One Health (OH) approach, which gained international prominence in the early 2000s and was formally articulated through a unified definition in 2022, promotes coordinated action across human, animal and environmental health sectors. Despite increasing global recognition, its integration into national health systems remains inconsistent. This review synthesizes global evidence on challenges, opportunities and strategic directions for embedding OH within health system structures.

**Study Design:**

A systematic review with narrative synthesis.

**Methods:**

This systematic review followed Preferred Reporting Items for Systematic Reviews and Meta‐Analyses (PRISMA) 2020 guidelines. Peer‐reviewed studies published between 1 June 2013, and 1 April 2025 were identified through searches conducted in January–February 2025 in Scopus, PubMed, Web of Science and ProQuest Health and Medicine. Supplementary searches included Google Scholar and backward reference screening. Two reviewers independently screened studies and applied predefined eligibility criteria. Findings were synthesized narratively.

**Results:**

Of 2606 records identified, 17 studies were included. The studies represented Africa, Europe, Asia, Australia and multi‐country settings across varying income levels. Key challenges included weak governance arrangements, limited legal and policy support, inadequate financing, workforce capacity constraints and persistent sectoral fragmentation. Opportunities were linked to heightened awareness of zoonotic threats and antimicrobial resistance, growing institutional endorsement, academic engagement and emerging multisectoral coordination mechanisms.

**Conclusion:**

Sustainable integration of OH requires embedding it within governance, financing and service delivery systems rather than implementing it as parallel initiatives.

## Graphical abstract

This systematic review synthesizes global evidence on the integration of One Health into national health systems. It identifies key system‐level barriers, including governance fragmentation, financing constraints, workforce limitations, and policy gaps, alongside emerging opportunities such as institutional support and multisectoral collaboration. The findings emphasize that sustainable One Health implementation requires structural embedding within health system functions rather than isolated initiatives.

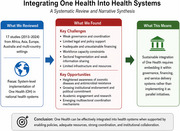

## 1 Introduction

A health system is defined as ‘all the organizations, people, and actions whose primary intent is to promote, restore, or maintain health’ [1]. Traditionally, health systems have focused largely on human health, whereas animal health and environmental management have been governed through parallel and often fragmented systems. One Health (OH) addresses this fragmentation and is defined as ‘an integrated, unifying approach that aims to sustainably balance and optimize the health of people, animals, and ecosystems’ [2]. Although the interconnection between human and animal health has long been acknowledged historically, the contemporary OH movement gained global prominence in the early 2000s and was formally consolidated through a unified definition developed by the One Health High‐Level Expert Panel in 2022 [2]. Rooted in long‐standing recognition of the interconnectedness of human, animal and environmental health, the OH approach seeks to strengthen coordination across sectors and embed collaborative action within existing health system structures. The OH approach addresses critical health challenges, including emerging and re‐emerging epidemics and pandemics, antimicrobial resistance, food safety and security, climate change and environmental contamination [3]. A landmark global analysis published in 2008 reported that approximately 60% of known infectious diseases and 75% of emerging infectious diseases are zoonotic in origin [4]. A 2011 World Health Organization (WHO) regional report highlighted that major outbreaks, such as H5N1 avian influenza, Ebola, H1N1 pandemic influenza, SARS and MERS‐CoV, were zoonotic in origin and linked to wildlife [5]. Additionally, antimicrobial resistance is accelerating at an alarming rate, necessitating a collaborative response across human, animal and environmental health sectors [6, 7, 8, 9]. Recent global burden estimates based on 2019 data and published in 2022 further demonstrate the accelerating impact of antimicrobial resistance [8].

Several global trends further emphasize the urgency of OH implementation, including rapid urbanization, globalization, ecosystem disturbances, increasing human and animal populations, climate change and escalating socio‐economic and food security crises [10, 11, 12, 13, 14, 15, 16, 17]. Despite its significance, OH remains largely unintegrated into national governance and service delivery structures, with limited coordination across human health, animal health and environmental sectors [14], and only a few countries demonstrating uneven progress toward cross‐sectoral integration [16]. For example, a 2020 study conducted in Uganda examined national‐level operationalization of OH and documented coordination gaps and institutional fragmentation [18].

Scholars have identified multiple factors hindering OH implementation at global, national, regional and local levels. A recurring challenge is limited awareness and understanding of OH among policymakers, health system leaders and frontline professionals, which constrains its incorporation into national human health, veterinary and environmental management systems [19, 20]. A study [11] reported that OH is often misunderstood by policymakers and professionals across human, animal and environmental sectors, who may perceive it as causing role overlap rather than enabling interdisciplinary collaboration [21]. Furthermore, disciplinary dominance within human health institutions, often marginalizing veterinary, environmental and wildlife perspectives, has been identified as a barrier to effective OH integration [22].

The implementation of the OH approach entails notable challenges but also offers substantial opportunities, as evidenced by several key studies. Frequently cited barriers include inadequate institutional structures, fragmented coordination across sectors, limited financial resources and weak political support—issues particularly pronounced in low‐ and middle‐income countries [16, 23]. These challenges are intensified by disciplinary fragmentation, ambiguous legal and operational frameworks, and difficulties in integrating cross‐sectoral knowledge and evaluation tools [24, 25]. Additionally, top‐down OH strategies often fail to reflect local realities, resulting in poor community engagement and overreliance on external support [26].

Despite these constraints, OH holds considerable promise for enhancing the prevention and control of zoonotic diseases, antimicrobial resistance and health impacts of climate change through integrated and multisectoral collaboration. Opportunities identified in the literature include utilizing existing institutional networks, encouraging community‐driven initiatives, adopting participatory policy development and applying systems‐thinking approaches to improve performance evaluation [23, 25]. Scholars recommend embedding OH principles into national public health, animal health, environmental protection and emergency preparedness policies; establishing supportive legal and educational frameworks; strengthening local leadership; and promoting cross‐disciplinary knowledge exchange to ensure sustainable and contextually relevant implementation.

Furthermore, OH is receiving increasing global endorsement. In 2022, the Quadripartite, comprising the WHO, the Food and Agriculture Organization of the United Nations (FAO), the World Organisation for Animal Health (WOAH) and the United Nations Environment Programme (UNEP), launched the One Health Joint Plan of Action (2022–2026) to guide coordinated implementation [2, 12, 27].

Funding agencies are increasingly prioritizing interdisciplinary programmes, recognizing the potential of OH to address complex and interconnected health challenges more effectively than isolated, sector‐specific approaches. Nevertheless, much of the empirical literature remains region‐specific. Country‐level assessments in Uganda [18], Nepal [28] and Somalia [29], for example, report progress in multisectoral collaboration while simultaneously identifying persistent barriers related to financing, coordination, governance and institutional sustainability. Although these studies provide valuable contextual insights, their findings are largely confined to individual national experiences.

More recent reviews published in 2025 [30, 31, 32] have examined operational challenges in rural African contexts, ethical considerations within a globalized OH framework and critical reflections from the Global South. Although these contributions broaden thematic and regional understanding, they primarily focus on conceptual or context‐specific dimensions rather than systematically analysing how implementation barriers and facilitators intersect with core health system functions.

As a result, there remains limited clarity on how governance arrangements, financing mechanisms, workforce capacity and institutional coordination collectively influence the integration of OH into national health systems. Implementation challenges frequently reflect systemic patterns that transcend national boundaries rather than isolated country experiences. A comparative synthesis is therefore necessary to identify recurring structural barriers and enabling factors across diverse contexts.

Accordingly, this systematic review synthesizes global evidence on the challenges and opportunities of integrating OH into national health systems across human, animal and environmental sectors and identifies actionable system‐level strategies to support sustainable integration.

## 2 Methods

This systematic review was conducted following the Preferred Reporting Items for Systematic Reviews and Meta‐Analyses (PRISMA) 2020 guidelines. The review protocol was registered in PROSPERO under registration number CRD42024520269. The completed PRISMA 2020 checklist, including page and line number references corresponding to each reporting item, is provided as Supporting Information S1.

### 2.1 Data Sources and Search Strategy

Studies were identified through online database searches and by reviewing reference lists of relevant articles. The search was conducted in Scopus, PubMed, Web of Science and ProQuest Health and Medicine using the following keywords: *One Health AND (Challenges OR Obstacles OR Barriers OR Defiance) AND (Opportunities OR Advantages OR Benefits OR Enablers) AND (Health Systems OR Health Programmes OR Health Services OR Health Strategies)*. The complete search strategies for each database, including Boolean operators and database‐specific adaptations, are provided in Supporting Information S2. In this review, the term *health system* was operationalized broadly to capture system‐level and implementation‐focused evidence. Search terms, such as *health programmes, health services* and *health strategies*, were intentionally included because OH is frequently implemented through specific programmes, service delivery arrangements and national or sub‐national strategies rather than explicitly labelled ‘health system’ reforms. Including these related terms enabled identification of relevant literature addressing governance, coordination and operational integration of OH across human, animal and environmental sectors. Additionally, Google Scholar was used as a supplementary search tool to identify relevant studies that may not be indexed in traditional bibliographic databases. For Google Scholar, results were screened by relevance ranking, and the first 200 records were exported and stored in reference management software to ensure reproducibility. Duplicate records were removed prior to screening. Following full‐text screening, the reference lists of all included studies were manually reviewed to identify additional eligible articles. The search strategy was developed in consultation with and reviewed by a professional health librarian to enhance methodological rigour and completeness. Corresponding authors of any literature identified were not contacted for additional information.

### 2.2 Inclusion and Exclusion Criteria

Eligibility criteria were structured using the PICo framework (population, interest and context), which is appropriate for non‐clinical and policy‐focused research questions:

- **Population (P)**: national health systems, institutions and stakeholders involved in OH implementation.
- **Interest (I)**: challenges, opportunities, barriers, facilitators and implementation strategies related to OH integration.
- **Context (Co)**: human, animal and environmental health sectors at national or sub‐national levels.

This review includes peer‐reviewed studies published between 1 June 2013, and 1 April 2025, examining the challenges and opportunities of OH implementation in health systems, programmes, services or strategies; the start date was selected to align with the period in which OH gained formal global policy recognition and began to be operationalized through national and international frameworks. Studies providing broader insights into OH implementation challenges and opportunities were also included, even when not explicitly focused on specific health systems or programmes, because they elucidate cross‐cutting governance, coordination and workforce dynamics that directly shape how OH is operationalized within human, animal and environmental health systems. Only peer‐reviewed journal articles published in English were included. Conference abstracts lacking full‐text availability were excluded due to insufficient methodological detail, empirical findings or policy‐relevant analysis required to extract implementation challenges, opportunities or recommendations. Grey literature was also excluded to ensure methodological consistency and comparability across included studies. Database searches were conducted between 10 February and 15 April 2025.

### 2.3 Selection and Screening Process

Two reviewers (M.S.I. and D.T.G.) independently screened titles and abstracts. Full‐text screening was conducted independently by the same reviewers, and disagreements were resolved through discussion with a third author (N.J.). All reviewers were members of the author team. Covidence was used to manage the screening workflow; however, no automated decision‐making tools or artificial intelligence systems were used during study selection.

### 2.4 Data Extraction and Management

Data extraction was performed independently by two reviewers (M.S.I. and D.T.G.) using a standardized extraction form. Discrepancies were resolved through discussion and consensus, with involvement of a third reviewer (N.J.) when necessary. A standardized data extraction form was developed to systematically collect relevant details from the included studies. Extracted data included publication type, geographical location, study setting, publication year, study duration, objectives, study design, sample size, data collection methods, key findings and limitations. During the whole process, there was no automated data extraction tool used.

### 2.5 Quality Assessment

The included studies were critically evaluated using separate assessment tools for primary studies and review articles:

- Primary studies were appraised using the Joanna Briggs Institute (JBI) critical appraisal checklist for cross‐sectional studies. Although no restrictions were placed on study design at the eligibility stage, the majority of included primary studies employed cross‐sectional or descriptive observational designs, which are common in OH implementation research. The JBI checklist was therefore selected as the most appropriate and consistently applicable tool to assess methodological quality across the included studies. The tool evaluated sample size adequacy, study participants and settings, use of standard criteria, identification and management of confounding factors, reliability of outcome measurement and appropriateness of statistical analysis. Responses were recorded as ‘Yes’, ‘No’, ‘Unclear’ or ‘Not applicable’, and studies were categorized as included, excluded or requiring further information (Supporting Information S3).
- Review articles were evaluated using the Critical Appraisal Skills Programme (CASP) Systematic Review Checklist (Supporting Information S4). This tool focused on study validity, key findings and applicability to local contexts, with responses categorized as ‘Yes’, ‘Cannot Tell’ or ‘No’.

The first author conducted the initial assessments, with the second author verifying each study for consistency. Any disagreements regarding risk of bias were resolved through discussion or consultation with a third author.

### 2.6 Data Synthesis

Data were synthesized narratively in alignment with the structure of the results section, beginning with study characteristics followed by thematic analysis of challenges, opportunities and recommendations. Challenges, opportunities and recommendations were identified through iterative coding of extracted data. Statements describing barriers, constraints or implementation difficulties were categorized as challenges; descriptions of enabling conditions or supportive factors were classified as opportunities; and proposed actions or policy suggestions were categorized as recommendations:

- The challenges and opportunities identified in each study were compiled in tables and textual descriptions.
- A narrative synthesis was conducted to interpret the findings, applying a thematic analysis to systematically identify, code and synthesize barriers and facilitators of OH implementation across health system domains within human, animal and environmental sectors.
- Recommended strategies for improving OH implementation were identified and analysed.
- General study characteristics (location, publication type, study design, data collection methods and sampling techniques) were summarized and tabulated for further interpretation.

Study characteristics were summarized in tabular format. Thematic findings were organized into structured tables to facilitate comparison across settings, and frequency patterns were described narratively to highlight recurring system‐level themes. This structured approach ensured a comprehensive analysis of OH implementation across diverse contexts.

## 3 Results

### 3.1 Search Results

**FIGURE 1:**
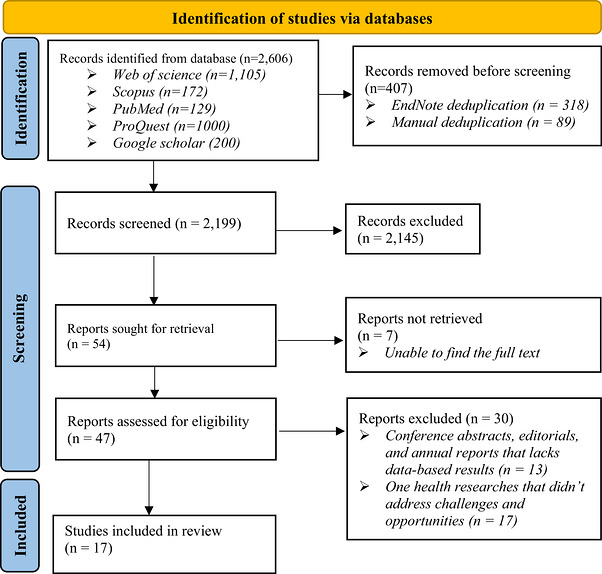
PRISMA diagram illustrating the process of identifying, screening and including studies for review.

The database search identified 2606 records, comprising 1105 from Web of Science, 1000 from ProQuest, 172 from Scopus, 129 from PubMed and 200 from Google Scholar (Figure 1). Following duplicate removal and title–abstract screening, a subset of studies advanced to full‐text assessment. After applying the predefined eligibility criteria, 17 studies were included in the final synthesis.

Seven records were excluded at the full‐text stage (Figure 1) due to inaccessibility; although abstracts were screened where available, they did not provide sufficient detail to determine eligibility in relation to system‐level challenges and opportunities.

Full‐text exclusions primarily comprise studies focusing on disease‐specific OH applications, laboratory‐based surveillance or conceptual discussions without empirical or policy‐relevant analysis of governance, financing, institutional integration or other system‐level implementation factors. These studies, published between 2013 and 2024 across Africa, Asia and Europe, largely addressed zoonotic disease surveillance, antimicrobial resistance monitoring or environmental risk assessment rather than systemic barriers or enabling mechanisms. Further details of the study selection process are presented in Figure 1.

### 3.2 Characteristics of Included Studies

**FIGURE 2:**
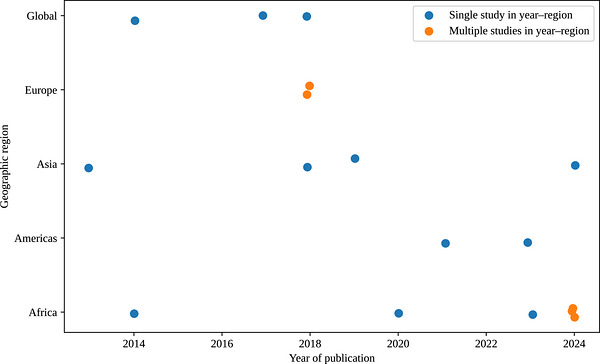
Geographic distribution and temporal evolution of included studies (2013–2024). Each point represents one study plotted by year of publication and geographic region. Minor positional adjustment was applied to avoid visual overlap of studies published in the same year and region.

The 17 included studies were published between 2013 and 2024, with an increase in publications observed after 2018. Geographically, studies represented diverse regions, including Sub‐Saharan Africa (e.g., Uganda, Somalia and Guinea), South Asia (e.g., Nepal and India), Southeast Asia, Europe, Australia, and multi‐country or global analyses. The geographic distribution and temporal evolution of the included studies are shown in Figure 2.

In terms of economic context, the majority of empirical studies were conducted in low‐ and middle‐income countries, whereas high‐income settings were primarily represented through policy analyses and cross‐national evaluations. Study designs included qualitative case studies, policy analyses, mixed‐methods research and systematic or scoping reviews (Table 1).

**TABLE 1:** Characteristics of included studies examining system‐level challenges and opportunities for integrating One Health (OH) into national health systems (*n* = 17).

Reference	Publication year	Country/Region	Income level	Study design	Study population/sample	Data collection methods	Primary focus
[33]	2013	South Asia (regional)	Mixed LMIC	Policy analysis	National stakeholders	Document review	Governance and coordination
[34]	2014	Global	Multi country	Conceptual analysis	Not applicable	Literature synthesis	Zoonotic complexity
[16]	2014	Sub‐Saharan Africa	LIC	Case analysis	Public health/veterinary sectors	Qualitative interviews	Institutional coordination
[23]	2018	Switzerland	HIC	Qualitative	Academic & policy actors	Interviews	Education and integration
[25]	2018	Europe	HIC	Programme evaluation	Universities	Mixed methods	Capacity building
[24]	2018	Global	Multi country	Conceptual review	Not applicable	Literature review	Systems framing
[35]	2018	China	UMIC	Policy analysis	National institutions	Document analysis	Surveillance integration
[28]	2019	Nepal	LMIC	Qualitative	National stakeholders	Interviews and document review	Governance and financing
[36]	2019	Multi country (Africa/Asia)	Mixed	Comparative analysis	National OH platforms	Policy and document review	Governance mechanisms
[18]	2020	Uganda	LIC	Case study	National OH platform	Interviews	Implementation barriers
[37]	2021	Caribbean	UMIC	Policy review	Regional institutions	Document review	Governance integration
[38]	2023	Ethiopia	LIC	Mixed methods	Health/veterinary sectors	Surveys and interviews	Infrastructure and workforce
[39]	2023	Latin America	MIC	Regional analysis	Multisector actors	Policy analysis	Institutional frameworks
[29]	2024	Somalia	LIC	Case study	Government stakeholders	Interviews	Institutionalization
[40]	2024	Ethiopia	LIC	Qualitative	National actors	Interviews	Policy and awareness
[41]	2024	India	LMIC	Mixed methods	Government & NGOs	Surveys/Interviews	Coordination challenges
[42]	2024	Ethiopia	LIC	Policy analysis	National framework	Document review	Legal and governance gaps

*Note:* Income level classification is based on the World Bank country income categories (2024 fiscal year). Countries are classified as low‐income (LIC), lower middle‐income (LMIC), upper middle‐income (UMIC) or high‐income countries (HIC). Multi‐country studies spanning countries across different income categories are labelled ‘mixed’ or ‘multi country’. Multi‐country studies include analyses conducted across more than one national context. Where country‐specific disaggregation was not provided, the study is categorized as ‘multi country/global’. Study population/sample refers to the primary actors or institutions examined in each study (e.g., national policymakers, public health officials, veterinary professionals and multisectoral coordination platforms). Data collection methods are presented separately from study population to enhance clarity, as recommended by peer review. Regional classification (e.g., Eastern Africa and South Asia) is based on the primary geographical focus reported in each study.

### 3.3 Quality Checks of the Included Studies

The methodological quality of the included primary studies was assessed using the JBI Critical Appraisal Checklist for cross‐sectional studies and the CASP tools for included review articles. Overall, most studies met key quality criteria related to clarity of objectives, appropriateness of study design and relevance of outcomes to OH implementation. Common methodological limitations included insufficient reporting on confounding factors, limited justification of sample size and variability in the reliability of outcome measurement. Detailed results of the critical appraisal for each included study are presented in Supporting Information S3 (JBI checklist) and Supporting Information S4 (CASP appraisal). These assessments informed the interpretation of findings but did not result in the exclusion of studies solely on the basis of quality.

### 3.4 Thematic Focus of Included Studies

The included studies addressed multiple dimensions of OH implementation. Thematically, studies focused on governance and coordination mechanisms, institutional integration, workforce development, financing structures, policy and regulatory frameworks, and multisectoral collaboration models. Several studies also examined disease surveillance, antimicrobial resistance and zoonotic disease preparedness within OH frameworks.

The studies were conducted with a variety of objectives related to OH implementation. Some focused on the challenges of OH implementation [28, 40, 42], whereas others examined awareness levels and the views of participants regarding OH [37, 41]. Some studies addressed OH implementation for zoonotic diseases [34, 36], whereas others explored implementation experiences [33] and analysed the state, prospects and difficulties of OH implementation [29]. Another study has focused on the conceptual and global evolution of OH [24], and others were targeted in the integration of knowledge in OH policy [23], the OH implementation situation of Africa [16] and systems approach to OH implementation [25]. Additionally, some studies discussed phage therapy as an alternative strategy for preventing food and waterborne diseases within an OH framework [39], the contribution of omics tools to OH implementation [38] and perspectives on antimicrobial use (AMU) and antimicrobial resistance (AMR) in the development of OH policy [35]. The included articles also highlighted the key challenges, opportunities and recommended actions for advancing OH implementation in specific contexts (Table 2).

**TABLE 2:** Thematic distribution of reported system‐level challenges in One Health implementation across included studies, categorized by income setting and health system domain.

Study	Income	Governance/Institutional fragmentation	Financing constraints	Workforce gaps	Policy/Legal gaps	Infrastructure/Surveillance	Awareness/Political commitment
[28]	LMIC	✓	✓	✓	✓		✓
[38]	LIC		✓	✓		✓	
[40]	LIC	✓			✓		✓
[41]	LMIC	✓					✓
[42]	LIC	✓			✓		
[29]	LIC	✓			✓		✓
[]	Multi	✓					✓
[16]	LIC	✓				✓	
[35]	UMIC	✓				✓	
[33]	LMIC			✓			✓
[39]	MIC	✓			✓		
[37]	UMIC	✓			✓		
[34]	Global					✓	
[23]	HIC	✓		✓			
[25]	HIC			✓			
[18]	LIC	✓	✓				✓
[24]	Global	✓					

*Note:* Challenge domains were categorized using a health systems analytical framework, including governance and institutional coordination, financing mechanisms, workforce capacity, policy and regulatory frameworks, infrastructure and surveillance systems, and political awareness or commitment. A check mark (✓) indicates that the study explicitly reported the corresponding challenge as a system‐level barrier to One Health integration. Income setting classification follows World Bank (2024) income group categories. Studies categorized as ‘multi country’ or ‘global’ include analyses that span multiple income levels or do not restrict findings to a single national context. Thematic coding was conducted through iterative extraction and synthesis of reported barriers described in the primary studies.

Abbreviations: HIC, high‐income countries; LIC, low‐income countries; LMIC, lower middle‐income countries; UMIC, upper middle‐income countries.

### 3.5 System‐Level Challenges in OH Implementation

#### 3.5.1 Governance and Institutional Fragmentation

Weak organizational structures and limited intersectoral coordination were consistently identified as major barriers to OH implementation [28, 39, 40, 41, 42]. Several studies reported insufficient cooperation among stakeholders and unclear institutional mandates, which constrained effective cross‐sectoral collaboration [36, 43]. In addition, gaps in awareness and political commitment among policymakers and key actors further limited coordinated action [28, 33, 34, 36, 40, 41, 44].

#### 3.5.2 Financing Constraints

Financial limitations were repeatedly reported as a structural bottleneck (Table 2). Insufficient and unstable budget allocations, coupled with limited domestic investment in OH initiatives, constrained implementation efforts across health systems [18, 28, 38]. The absence of dedicated financing mechanisms further hindered long‐term sustainability.

#### 3.5.3 Workforce Capacity and Human Resources

Workforce‐related constraints were prominent across studies (Table 2). A shortage of trained interdisciplinary professionals limited cross‐sectoral training opportunities, and inadequate technical expertise were identified as major impediments [28, 38]. Low levels of understanding of OH principles among political leaders and key actors also weakened implementation capacity [28, 33, 41].

#### 3.5.4 Policy and Regulatory Gaps

Inadequate legislative frameworks and weak regulatory structures were reported as systemic challenges limiting institutionalization of OH [28, 39, 40, 41, 42]. The absence of formalized policy mandates for multisectoral coordination contributed to fragmented implementation (Table 2).

#### 3.5.5 Infrastructure, Surveillance and Technological Integration

Infrastructure limitations, including weak laboratory systems and limited surveillance capacity, were identified as barriers to operationalizing OH [16, 38, 44] (Table 2). The integration of emerging technologies, such as OMICS and health informatics, was further constrained by high implementation costs, substantial infrastructure requirements and shortages of trained personnel [38].

Additionally, the inherent complexity of zoonotic diseases, driven by genetic, environmental, ecological, climatic and socio‐economic determinants combined with insufficient policy attention, complicated prevention and control efforts within OH frameworks [34].

### 3.6 Enabling Factors and Opportunities for Integration

Despite the identified barriers, multiple enabling factors were reported (Supporting Information S5). These included increasing global recognition of zoonotic threats and antimicrobial resistance [28, 33], institutional endorsement by international organizations [28, 29, 33], expansion of interdisciplinary academic programmes [29, 40] and the development of multisectoral coordination platforms [37, 41]. Several studies also noted growing political commitment and regional collaboration frameworks as catalysts for progress [34, 36]. Finally, the rising awareness levels among actors and stakeholders [37, 40] highlight an important shift toward greater support for OH implementation.

### 3.7 Reported Recommendations for Strengthening OH

The included studies proposed several strategies to advance OH integration (Supporting Information S5). These included formalizing governance structures and strengthening legal and policy frameworks [28, 39, 40, 41, 42], establishing dedicated and sustainable financing mechanisms [28, 38], and strengthening interdisciplinary workforce training and technical capacity development [28, 33, 38]. Several studies also emphasized the need to institutionalize cross‐sectoral coordination platforms and enhance multisectoral collaboration mechanisms [36, 41, 44]. In addition, integrating OH principles into national health policies and regulatory frameworks was identified as critical for sustainable implementation [39, 40, 42], alongside the development of standardized monitoring and evaluation systems to assess progress and ensure accountability [28, 36].

Some studies further recommended embedding OH within existing health system building blocks, such as governance, financing, service delivery and surveillance rather than implementing it as parallel or project‐based initiatives, emphasizing sustainability and long‐term institutionalization [28, 36, 38, 40].

## 4 Discussion

### 4.1 Overview of Study Aims and Key Findings

This systematic review synthesizes global evidence on the challenges and opportunities associated with integrating OH into national health systems across human, animal and environmental sectors. OH operates within the shared agendas of animal, human and environmental health [21], encompassing zoonoses, AMU and AMR, food safety and security, disease surveillance, governance, neglected and non‐communicable diseases, and socio‐economic determinants of health [45]. Although the concept of OH was introduced in the mid‐1800s, this review demonstrates that its institutional integration remains in an early developmental phase in many regions.

Across the 17 included studies, five interrelated system‐level domains emerged: governance and institutional coordination, financing and sustainability, workforce capacity and interdisciplinary expertise, policy and regulatory frameworks, and infrastructure and technological integration. These domains collectively shape the pace and sustainability of OH implementation.

### 4.2 Governance and Institutional Integration

Governance fragmentation was one of the most consistently reported barriers. Poor organizational structures, lack of formal OH institutions, absence of unified metrics and gaps in intersectoral collaboration were repeatedly highlighted [24, 25, 28, 41, 42]. Weak institutional coordination limits scalability and contextual adaptation of national OH strategies, as observed in Uganda [18].

In many settings, veterinary, public health and environmental sectors continue to operate independently, reflecting entrenched disciplinary silos [16, 23]. Legal ambiguities and absence of formal mandates further weaken cross‐sector collaboration [42]. These findings align with broader global analyses indicating that institutional integration remains one of the most complex dimensions of OH operationalization [12]. Sustainable OH integration requires formalized governance structures and legally anchored multisectoral coordination mechanisms.

### 4.3 Financing and Sustainability

Limited and unstable funding was identified as a major bottleneck across multiple settings [28, 29, 38]. In several low‐income countries, OH initiatives depend heavily on NGO or donor support, raising concerns regarding long‐term sustainability [29]. The absence of dedicated financing mechanisms prevents institutionalization within national health systems.

These findings are consistent with health systems research emphasizing that integration cannot be sustained without domestic budgetary allocation and financial accountability mechanisms [45]. The reliance on short‐term project funding limits structural embedding of OH activities. Without stable financing structures embedded in national budgets, OH initiatives risk remaining fragmented and externally driven.

### 4.4 Workforce Capacity, Ethics and Interdisciplinary Practice

Workforce limitations emerged as both structural and cultural barriers. Constraints related to trained manpower, limited interdisciplinary expertise and insufficient political understanding of OH were reported [28, 33, 38]. The awareness gap identified in 2013 [33] remains evident in later studies [34], suggesting slow progress in institutional adoption.

Research ethics procedures were also identified as significant obstacles. The lack of OH‐accredited institutions and multidisciplinary ethics review protocols frequently results in denial or delay of research approval, particularly in African contexts [40]. Multisectoral studies often require approval from multiple ethics committees, creating administrative inconsistencies [46]. Developing harmonized OH‐specific ethics guidelines could streamline research while safeguarding multidisciplinary integrity.

Professional resistance also influences implementation. Medical professionals have shown comparatively greater reluctance to adopt OH frameworks than veterinary or environmental health experts [37]. Such resistance may reflect professional hierarchies and lack of accreditation systems. Strengthening interdisciplinary education, harmonizing research ethics procedures and institutionalizing OH accreditation are critical to advancing professional integration.

### 4.5 Policy, Legal Frameworks and Infrastructure

The absence of legal frameworks and standardized policy guidelines was repeatedly cited as a barrier [34, 41, 42]. In many settings, OH initiatives operate without formal legislative support, limiting authority and accountability.

Infrastructure gaps further constrain implementation. Limited laboratory capacity, fragmented surveillance systems, weak data‐sharing practices and lack of standardized organizational indicators impede coordinated response [36, 42]. The prioritization of zoonotic diseases remains insufficient despite evidence that 60% of known zoonoses and 75% of emerging infectious diseases originate from animals [45].

Emerging technologies, such as omics, metabolomics and bioinformatics, offer significant potential but are constrained by high costs, need for specialized infrastructure and shortage of trained professionals [38]. The example of bacteriophage therapy illustrates how regulatory, laboratory, workforce and legislative barriers collectively delay system‐wide adoption [39]. Policy formalization and infrastructure investment are mutually reinforcing prerequisites for effective OH institutionalization.

### 4.6 Opportunities and Enabling Factors

Despite these challenges, several enabling factors were identified. Major epidemics, including avian influenza, Ebola and COVID‐19 have accelerated political recognition of OH [28, 33, 36, 42]. Persistent endemic challenges such as rabies and antimicrobial resistance further motivate multisectoral collaboration [28].

Institutional endorsement from WHO, FAO, WOAH and UNEP has strengthened global commitment [2, 12]. Funding agencies increasingly prioritize interdisciplinary programmes. Community‐driven and systems‐thinking approaches enhance contextual adaptation [23, 25, 26].

Academic institutions play a pivotal role in establishing OH platforms, developing communication strategies, prioritizing zoonotic diseases and advancing multidisciplinary research [28, 29, 34]. Innovations such as cloud technologies and metabolomic profiling further strengthen implementation capacity [38]. Growing institutional commitment and technological advancement provide a strategic window for embedding OH within national health systems.

### 4.7 Representativeness and Characteristics of Included Studies

The included studies represent diverse geographic and income settings, with concentration in Sub‐Saharan Africa and South Asia. Most empirical studies were conducted in low‐ and middle‐income contexts, where zoonotic burdens are substantial. High‐income settings were more frequently represented through conceptual or educational analyses.

The predominance of cross‐sectional and qualitative designs may influence interpretation as such approaches provide contextual depth but limited longitudinal insight. Nevertheless, thematic convergence across settings strengthens confidence in the synthesized findings.

## 5 Strengths and Limitations

### 5.1 Strengths

This review has several methodological and evidentiary strengths that enhance the validity and credibility of its findings. The study followed PRISMA 2020 reporting guidelines, ensuring transparency in study identification, screening, eligibility assessment and synthesis. Multiple databases were searched over a publication period (2013–2024) that corresponds with the growing institutionalization of the OH agenda, enabling inclusion of both early conceptual discussions and more recent implementation‐focused analyses. The application of a structured health systems framework allowed systematic categorization of governance, financing, workforce, policy and infrastructure dimensions across diverse contexts. Inclusion of studies from varied income settings strengthens the global relevance of the findings. Moreover, the convergence of themes across heterogeneous methodological designs (qualitative, policy analysis and mixed methods) increases confidence in the robustness of the synthesized patterns.

### 5.2 Limitations

Despite these strengths, several limitations should be acknowledged.

#### 5.2.1 Language Restriction

Only English‐language peer‐reviewed publications were included. This may have excluded relevant evidence published in other languages, particularly from regions where OH initiatives are emerging, but academic dissemination occurs in local languages. The dominance of English in global scientific publishing has been shown to disadvantage researchers from non‐English‐speaking countries, potentially limiting the visibility of locally generated evidence [47]. This may result in underrepresentation of certain regional experiences within the synthesized findings. However, most internationally indexed OH research is published in English, which partially mitigates this limitation. Future reviews could incorporate multilingual search strategies or regional databases to enhance inclusivity and representation.

#### 5.2.2 Exclusion of Grey Literature

Institutional reports, governmental policy documents and technical briefs were excluded to maintain methodological rigour and comparability across studies. Although this strengthens internal validity and reproducibility, it may have limited inclusion of operational insights from national OH platforms that are not disseminated through peer‐reviewed channels. To mitigate this, the review focused on rigorously reviewed publications to ensure transparency of methods and findings. Future complementary scoping reviews incorporating grey literature could provide additional implementation‐level perspectives.

#### 5.2.3 Heterogeneity of Study Designs and Contexts

The included studies employed diverse methodological approaches and were conducted across varied socio‐political settings, limiting direct comparability and causal inference. This heterogeneity also made bias assessment challenging, as no single appraisal tool was suitable for all study types. To address this, two complementary bias assessment tools were applied—JBI and CASP—on the basis of study design. Although this approach strengthened methodological rigour, limitations remain in achieving fully consistent bias evaluation across heterogeneous studies. A structured health systems framework was used to standardize thematic synthesis. Future research should adopt more harmonized and comparative designs to enhance bias assessment precision and generalizability.

#### 5.2.4 Reliance on Reported Findings

The synthesis depended on how challenges and opportunities were described in the primary publications. If certain institutional or political dynamics were underreported, they may be correspondingly underrepresented in this review. Independent screening and data extraction were conducted to reduce interpretive bias. Future primary research should explicitly document system‐level governance, financing and coordination dimensions to enhance evidence transparency.

#### 5.2.5 Potential Screening Constraints

Although a structured screening and reference‐checking process was implemented, relevant studies may have been overlooked if system‐level challenges were not clearly articulated in titles or abstracts [48]. Full‐text assessment and backward citation tracking were conducted to minimize this risk; however, expanded citation network analysis in future reviews may further reduce the possibility of omission.

## 6 Conclusion

This systematic review synthesized global evidence on the challenges and opportunities associated with integrating OH into national health systems. Across diverse geographic and income settings, five interrelated system‐level domains consistently shaped OH implementation: governance and institutional coordination, financing and sustainability, workforce capacity and interdisciplinary expertise, policy and regulatory frameworks, and infrastructure and technological integration.

The findings demonstrate that OH implementation remains structurally constrained by fragmented governance arrangements, unstable financing mechanisms, limited interdisciplinary workforce capacity, weak legal mandates, and inadequate surveillance and data‐sharing systems. These barriers are not isolated to specific countries but reflect systemic patterns that transcend national contexts. At the same time, increasing political organization of zoonotic threats, antimicrobial resistance and emerging infectious diseases, alongside institutional endorsement from global organizations, provides a strategic opportunity to embed OH within core health system functions.

Importantly, this review highlights that sustainable OH integration requires formalized multisectoral governance structures, dedicated financing frameworks, harmonized ethics and accreditation systems, strengthened laboratory and surveillance infrastructure, and standardized monitoring and evaluation mechanisms. Without structural embedding within existing health system building blocks, OH risks remaining a project‐based or externally driven initiative.

### 6.1 Recommendations

On the basis of synthesized evidence, several key recommendations emerge as follows:

- **For routine practice**: Establish and institutionalize multisectoral coordination platforms with clearly defined mandates and shared accountability mechanisms.
- **For policy**: Integrate OH principles into national legislation, allocate dedicated budget lines for OH activities and develop harmonized ethics review and accreditation frameworks to facilitate multidisciplinary research and implementation.
- **For research**: Conduct longitudinal and comparative implementation studies to evaluate outcomes of OH integration; expand representation from underrepresented regions and non‐English contexts and develop standardized indicators to assess system‐level integration.

Recent global analyses emphasize persistent challenges related to governance fragmentation, financing sustainability and operational coordination, reinforcing the need for system‐level reform rather than ad hoc collaboration. Aligning national OH strategies with such global guidance may strengthen coherence and long‐term impact.

In conclusion, advancing OH requires moving beyond conceptual endorsement toward structured, legally anchored and financially sustained integration within national health systems. The convergence of global political commitment and technological innovation provides a timely opportunity to institutionalize OH as a foundational framework for addressing complex health challenges in an interconnected world.

## Author Contributions

**Nusrat Jahan**: data curation, conceptualization, investigation, methodology, project administration, supervision, validation, visualization, writing – review and editing. **Md. Shahidul Islam**: conceptualization, data curation, formal analysis, funding acquisition, investigation, methodology, visualization, resources, writing – original draft, writing – review and editing. **Daniel Teshome Gebeyehu**: conceptualization, data curation, formal analysis, investigation, methodology, software, supervision, validation, visualization, writing – review and editing.

## Funding

The authors have nothing to report.

## Ethics Statement

The authors have nothing to report.

## Consent

The authors have nothing to report.

## Conflicts of Interest

The authors declare no conflicts of interest.

## Registration and Protocol

The systematic review protocol was registered in PROSPERO under registration number CRD42024520269.

## Supporting information




**Supporting Information S1:** PRISMA 2020 checklist: Completed PRISMA 2020 reporting checklist indicating page and line numbers corresponding to each reporting item addressed in the manuscript.

**Supporting Information S2**: Database‐specific search strategies: Comprehensive search strategies for each database, including full Boolean strings, keywords, MeSH terms (where applicable), date ranges and filters applied.

**Supporting Information S3**: JBI critical appraisal checklist for analytical cross‐sectional studies: Completed Joanna Briggs Institute (JBI) appraisal tool used to assess methodological quality and potential bias in cross‐sectional studies included in the review.

**Supporting Information S4**: CASP systematic review checklist: Completed Critical Appraisal Skills Programme (CASP) checklist used to assess the methodological rigour of included systematic reviews and analytical studies, where applicable.

**Supporting Information S5**: Detailed thematic extraction table: Comprehensive extraction matrix summarizing reported challenges, opportunities and recommendations across included studies, categorized by health system domain and study characteristics.

## Data Availability

The authors affirm that the data underpinning the findings of this study are presented in the results section of the article. Additional information regarding the data can be obtained from the corresponding author [M.S.I.], upon reasonable request.
