# Supplementary material for: Integrating One Health Into Health Systems: A Systematic Review and Narrative Synthesis of Implementation Challenges, Opportunities and Strategic Directions
Source: Public Health Chall. 2026 Apr 28;5(2):e70260. doi: 10.1002/puh2.70260 (PMC13123452; doi:10.1002/puh2.70260)
Supplement: Supplementary file 2 — Supporting Information S2: Database‐specific search strategies: Comprehensive search strategies for each database, including full Boolean strings, keywords, MeSH terms (where applicable), date ranges and filters applied. [file PUH2-5-e70260-s001.pdf]

Search strategies for each database used in the review.

1. PubMed

((((((((((((“One Health”[Title/Abstract]) AND (Challenges[Title/Abstract])) OR (Obstacles[Title/Abstract])) OR (Barriers[Title/Abstract])) OR (Defiance[Title/Abstract])) AND (Opportunities[Title/Abstract])) OR (Advantages[Title/Abstract])) OR (Benefits[Title/Abstract])) OR (Enablers[Title/Abstract])) AND ("Health Systems"[Title/Abstract])) OR ("Health Programs"[Title/Abstract])) OR ("Health Services"[Title/Abstract])) OR ("Health Strategies"[Title/Abstract]) AND ((ffrft[Filter]) AND (english[Filter]))

2. Scopus

(TITLE-ABS-KEY ("One Health") AND TITLE-ABS-KEY (Challenges) OR TITLE-ABS-KEY (Obstacles) OR TITLE-ABS-KEY (Barriers) OR TITLE-ABS-KEY (Defiance) AND TITLE-ABS-KEY (Opportunities) OR TITLE-ABS-KEY (Advantages) OR TITLE-ABS-KEY (Benefits) OR TITLE-ABS-KEY (Enablers) AND TITLE-ABS-KEY ("Health Systems") OR TITLE-ABS-KEY ("Health Programs") OR TITLE-ABS-KEY ("Health Services OR Health Strategies")) AND PUBYEAR > 2012 AND PUBYEAR < 2025 AND (LIMIT-TO (LANGUAGE , "English"))

3. Web of Science

“One Health” (Title) AND Challenges (Title) OR Obstacles (Title) OR Barriers (Title) OR Defiance (Title) AND Opportunities (Title) OR Advantages (Title) OR Benefits (Title) OR Enablers (Title) AND “Health Systems” (Title) OR “Health Programs” (Title) OR “Health Services OR Health Strategies” (Title) and 2025 or 2024 or 2023 or 2022 or 2021 or 2020 or 2019 or 2018 or 2017 or 2016 or 2015 or 2014 or 2013

4. ProQuest Health and Medicine

title("One Health") AND title(Challenges) OR title(Obstacles) OR title(Barriers) OR title(Defiance) AND title(Opportunities) OR title(Advantages) OR title(Benefits) OR title(Enablers) AND title("Health Systems") OR title(“Health Programs”) OR title(“Health Services OR Health Strategies”): From 2013 to 2025; Article, Interview, Literature Review, Review; English
