# Supplementary material for: Integrating One Health Into Health Systems: A Systematic Review and Narrative Synthesis of Implementation Challenges, Opportunities and Strategic Directions
Source: Public Health Chall. 2026 Apr 28;5(2):e70260. doi: 10.1002/puh2.70260 (PMC13123452; doi:10.1002/puh2.70260)
Supplement: Supplementary file 3 — Supporting Information S3: JBI critical appraisal checklist for analytical cross‐sectional studies: Completed Joanna Briggs Institute (JBI) appraisal tool used to assess methodological quality and potential bias in cross‐sectional studies included in the review. [file PUH2-5-e70260-s004.pdf]

Additional file 1. Quality check of primary research articles using Joanna Briggs Institute (JBI) Critical Appraisal Checklist for Cross-Sectional Studies

**Checklists**	Belaynehe *et al*., 202	Bera *et al*., 2024	Hassan-Kadle *et al*., 2024	Roopnarine, 2021	Standley *et al*., 2017	Tegegne *et al*., 2024	Zhuo *et al*., 2018
Were the criteria for inclusion in the sample clearly defined?	Yes	Yes	Yes	Yes	Yes	Yes	Yes
Were the study subjects and the setting described in detail?	Yes	Yes	Yes	Yes	Yes	Yes	Yes
Was the exposure measured in a valid and reliable way?	Yes	Yes	Yes	Yes	Yes	Yes	Yes
Were objective, standard criteria used for measurement of the condition?	Yes	Yes	Yes	Yes	Yes	Yes	Yes
Were confounding factors identified?	Unclear	Yes	Yes	Unclear	Unclear	Unclear	Yes
Were strategies to deal with confounding factors stated?	Unclear	Unclear	Unclear	Unclear	Unclear	Unclear	Yes
Were the outcomes measured in a valid and reliable way?	Yes	Yes	Yes	Yes	Yes	Yes	Yes
Was appropriate statistical analysis used?	Yes	Yes	Yes	Yes	Yes	Yes	Yes
**Decision**	Included	Included	Included	Included	Included	Included	Included
