# Supplementary material for: Integrating One Health Into Health Systems: A Systematic Review and Narrative Synthesis of Implementation Challenges, Opportunities and Strategic Directions
Source: Public Health Chall. 2026 Apr 28;5(2):e70260. doi: 10.1002/puh2.70260 (PMC13123452; doi:10.1002/puh2.70260)
Supplement: Supplementary file 4 — Supporting Information S4: CASP systematic review checklist: Completed Critical Appraisal Skills Programme (CASP) checklist used to assess the methodological rigour of included systematic reviews and analytical studies, where applicable. [file PUH2-5-e70260-s005.pdf]

Additional file 2. Quality check of review articles based on Critical Appraisal Skills Program (CASP) Systematic Review Checklist

**Checklists**	Acharya *et al*., 2019	Buregyeya *et al*., 2020	Gebreyes *et al*., 2014	Gongal, 2013	Lavilla *et al*., 2023	Tigistu-Sahle *et al*., 2023
Did the review address a clearly focused question?	Yes	Yes	Yes	Yes	Yes	Yes
Did the authors look for the right type of papers?	Yes	Yes	Yes	Yes	Yes	Yes
Do you think all the important, relevant studies were included?	Can’t tell	Can’t tell	Can’t tell	Can’t tell	Can’t tell	Can’t tell
Did the review’s authors do enough to assess quality of the included studies?	Yes	Yes	Yes	Yes	Yes	Yes
If the results of the review have been combined, was it reasonable to do so?	N/A	N/A	N/A	N/A	N/A	N/A
Can the results be applied to the local population?	Yes	Yes	Yes	Yes	Yes	Yes
Were all important outcomes considered?	Yes	Yes	Yes	Yes	Yes	Yes
Are the benefits worth the harms and costs?	Yes	Yes	Yes	Yes	Yes	Yes
**Decision**	Included	Included	Included	Included	Included	Included

N/A=Not applicable; Can’t tell= Can not tell
