# Supplementary material for: Integrating One Health Into Health Systems: A Systematic Review and Narrative Synthesis of Implementation Challenges, Opportunities and Strategic Directions
Source: Public Health Chall. 2026 Apr 28;5(2):e70260. doi: 10.1002/puh2.70260 (PMC13123452; doi:10.1002/puh2.70260)
Supplement: Supplementary file 5 — Supporting Information S5: Detailed thematic extraction table: Comprehensive extraction matrix summarizing reported challenges, opportunities and recommendations across included studies, categorized by health system domain and study characteristics. [file PUH2-5-e70260-s003.pdf]

Supplementary Table S3. Detailed thematic extraction of challenges, opportunities, and recommendations.

Studies' focus areas	Authors	Identified challenges	Identified opportunities	Studies' recommendations
Acharya et al., 2019	OH, implementation challenges and available opportunities	♦ Lack of OH organizational structure<br>♦ Lack of legal framework and implementation policy for OH<br>♦ Absence of collaboration among government agencies and institutions<br>♦ Lack of trained manpower<br>♦ Limited data sharing practice among sectors<br>♦ Budget constraints and low-level understanding of politician	♦ Emergence of multidisciplinary health challenges like AMR, highly pathogenic Avian Influenza, and Rabies enforces to use OH approach<br>♦ Engagement of NGOs in supporting OH and advocating its significance	♦ OH, legal frameworks should be created<br>♦ Detailed OH awareness should be created for politicians, policy makers and professionals<br>♦ Adequate budget should be allocated for the implementation of OH
Belaynehe et al., 2024	OH implementation challenges faced by scholars, RECs, and regulatory bodies in Africa	♦ Insufficient proposal review time<br>♦ Inadequate knowledge for OH research<br>♦ Lack of emergency protocols<br>♦ Insufficient knowledge of ethical issues specific to OH<br>♦ Lack of legal framework<br>♦ Lack of multidisciplinary review board	♦ Creation of multidisciplinary committee<br>♦ Formation of mandatory OH review strategies<br>♦ Formulation of standard OH proposal review procedure<br>♦ Awareness creation for REC members<br>♦ Creation of OH communications channels among researchers	♦ OH and emergency research ethics review strategies needs to be timely reviewed and implemented.
Bera et al., 2024	Determining awareness levels, attitudes on collaboration, and perspectives among OH collaboration	♦ Lack of established guidelines<br>♦ Insufficient political focus<br>♦ Limited cooperation among departments<br>♦ Lack of community awareness	♦ Stakeholders understood that intersectoral coordination is crucial for the operationalization of OH	♦ By establishing standard guidelines and guaranteeing stakeholder coordination, OH operationalization could be improved.
Buregyeya et al., 2020	Describing achievements of OH, identifying its challenges, and making recommendations on implementations of OH	♦ Insufficient multisectoral integration<br>♦ In adequate commitment from the government<br>♦ Gaps in awareness creation, advocacy, and research	♦ Establishment of national OH platform<br>♦ Memorandum of understanding among sectors<br>♦ Listing of priority zoonotic diseases<br>♦ OH Strategic Plan and communication strategy<br>♦ Activities of academia in OH integration	♦ Urgent steps should be taken, with government support, to address present and future issues to ensure systematic and sustainable OH involvement.

Destoumieux-Garzon et al., 2018	Conceptual and global evolution of OH	♦ Ambiguity in scope- Fragmented disciplines- Legal and operational gaps	♦ OH as a framework for AMR, zoonoses, and environmental threats- Global support from institutions (e.g., WHO, FAO, OIE)	♦ Institutionalize OH in policies- Foster interdisciplinary education and research- Create enabling legal frameworks
Gebreyes et al., 2014	Highlighting developments in priority zoonotic disease and OH capacity needs.	♦ Zoonotic diseases are complex and related with different genetic, anthropogenic, environmental, ecologic, climatic and socioeconomic factors<br>♦ Lack of concern to zoonotic diseases, especially in less developed regions<br>♦ Presence of integral pushing factors that make difficult to predict and prevent zoonotic diseases	♦ Notable advancements have been made in clinical diagnostic techniques, medical practices, and diseases surveillance systems	♦ Establishing science-oriented risk management guidelines<br>♦ Skilled manpower capacity building<br>♦ Accredited multidisciplinary diagnostic laboratories<br>♦ Equitable use of existing resources
Gongal, 2013	Addressing the experiences of regional countries to address and early prepared for emerging disease threats.	♦ Dominancy and ownership related conflicts<br>♦ Crossing professional boundaries<br>♦ Awareness gaps<br>♦ Lack of political commitment	♦ Multi-disciplinary and multifactorial diseases outbreaks like avian influenza, Severe Acute Respiratory Syndrome, Nipah virus and Ebola<br>♦ Presence of shared tasks that necessitates multidisciplinary involvement (AMR, Zoonotic diseases, food safety and security)<br>♦ Establishment of common frameworks<br>♦ Global warming, population growth, increased global trafficking and farming systems<br>♦ Involvement of NGOs in OH implementation	♦ Even if OH is becoming more widely recognized, but it still needs to be put into practice through national initiatives that are pertinent circumstances.
Hassan-Kadle et al., 2024	Describing the state, prospects, and difficulties of OH in Somalia and offering suggestions for institutionalizing and advancing it	♦ Insufficient finances and facilities<br>♦ Inadequate institutional capability and governance	♦ Somalia is involving various external funding OH initiatives<br>♦ Projects are currently working on research, capacity development, and community interventions<br>♦ Establishment of University initiative like Somalia OH Centre.	♦ The government is suggested to institutionalize and implement OH action plans from federal to district administrative levels
Hitziger et al., 2018	Knowledge integration in OH policy	♦ - Difficulty integrating knowledge across disciplines- Divergent	♦ - Better policy design using multisectoral knowledge-	♦ Establish knowledge-sharing platforms- Use participatory

		institutional priorities- Lack of metrics	Strengthening evidence-informed decisions	approaches in policy design- Develop common evaluation indicators
Lavilla et al., 2023	Examining the use of phages to prevent foodborne and waterborne diseases in a OH platform	♦ Extreme specificity of phages<br>♦ Potential phage resistance formation<br>♦ Phage stability and administration routes<br>♦ Legislative approval<br>♦ Consumer acceptance<br>♦ Resistant gene transmission among bacteria<br>♦ Phage biobanking for immediate trials	♦ An excellent antimicrobial alternative<br>♦ Self-dosage<br>♦ Effective to use phages in industries like food safety	♦ Future research ought to address the outstanding problems and primary hurdles to support phage-based practical use in the future
Okello et al., 2014	OH implementation in Africa	♦ Weak institutional frameworks- Low political support- Sectoral silos- Limited resources	♦ Potential for integrated zoonotic disease control- Platform for intersectoral dialogue- Use of existing networks	♦ Build sustainable policy frameworks- Promote long-term intersectoral collaborations- Support locally driven OH capacity
Roopnarine & Regan, 2021	Examining the views of academics in the medical, veterinary, and public health fields regarding the necessity, opportunities, and challenges of OH curriculum	♦ Absence OH accreditation system in the medical curriculum<br>♦ Resistance of accepting OH among the medical educators	♦ OH is recognised as a critical concept<br>♦ OH is the best approach for shared learning and research, more importantly in interprofessional education	♦ Strategies for convincing medical educators and students to accept OH in the curriculum and encourage a collaborative learning culture are among the implications for practice.
Ruegg et al., 2018	Systems approach to OH evaluation	♦ Inconsistent evaluation tools- Sectoral fragmentation- Poor communication across disciplines	♦ Systems thinking to assess performance- Common language and framework to assess collaboration	♦ Apply systems-based tools (e.g., NEOH framework)- Train stakeholders in evaluation methods- Align goals and outcomes across sectors
Standley et al., 2019	Determine and evaluate the structures and procedures currently in place for the detection and management of zoonotic diseases	♦ Coordination gaps among key actors<br>♦ Lack of continues training to stakeholders<br>♦ Lack of adequate infrastructure<br>♦ Public awareness gaps<br>♦ Extended research gaps on zoonoses	♦ Exiting linkage among top level ministries are encouraging<br>♦ New OH platform is launched<br>♦ Excellent future OH financial commitment of ministries	♦ Adequate investment and fulfilment of resources are needed for the timely implementation of OH

		♦ Lack of OH sustainability when the funded project ends		
Tegegne et al., 2024	Summarizing the takeaways from the assessment of OH surveillance, the challenges in implementing OH, the obstacles that impede OH functioning, and potential fixes for them.	♦ Poor organization and performance of OH surveillance systems<br>♦ Lack of operational and shared leadership<br>♦ Lack of adherence to fair data principles<br>♦ Limited sharing of methodologies<br>♦ Absence of standardized indicators	♦ Continuing health complexities are demanding multidisciplinary (One Health)	♦ Creating a formal governance board comprising members from every industry could help to remove obstacles that have existed for a while.<br>♦ It might be easier to establish OH surveillance systems if the effects of surveillance's OH-ness are demonstrated.
Tigistu-Sahle et al., 2023	Providing an overview of omics tools and their possible applications in OH applications in tropical diseases	♦ The cost of genomics is expensive, and while the cost of metagenomics is more escalation.<br>♦ Bioinformatics needs trained manpower<br>♦ Bioinformatics is an analytical tool that needs sophisticated infrastructure and storage<br>♦ Lack of skilled manpower and necessary infrastructure<br>♦ Lack of proper and multidimensional statistical software	♦ Internet and cloud technologies enables remote learning and data analysis<br>♦ The advancement of metabolomic profiling and metabolomic fingerprinting for OH applications<br>♦ Metabolomic application in food safety and toxicity, non-communicable disease and environmental health has a synergetic effect for OH application	♦ Capacity building in the tropics is critical to achieving equitable global health.<br>♦ Improving laboratory infrastructure and personal capacity building are crucial
Zhuo et al., 2018	Examine and contrast the information, perspectives, and experiences of health professionals AMU and AMR and exploring OH policy development.	♦ Externalizing of responsibilities among different medical professionals (Veterinary, Medical doctors, and dentists)<br>♦	♦ Prescribers are open to accept OH approach<br>♦ Professionals are aware about the ever-increasing antimicrobial resistance	♦ When developing policies, consideration should be given to obstacles unique to prescriber groups<br>♦ It will be necessary to develop and introduce interventions with caution in case they are seen as diminishing prescriber autonomy

OH = One Health; NGOs = None-Governmental Organization; REC = Research Ethics Committee; AMR = Antimicrobial Resistance; AMU = Antimicrobial Use
